# Q-Bot: automatic DICOM metadata monitoring for the next level of quality management in nuclear medicine

**DOI:** 10.1186/s40658-021-00371-w

**Published:** 2021-03-18

**Authors:** Ferenc Nagy, Aron K. Krizsan, Kornél Kukuts, Melinda Szolikova, Zsolt Hascsi, Sandor Barna, Antonietta Acs, Peter Szabo, Lajos Tron, Laszlo Balkay, Magnus Dahlbom, Mihaly Zentai, Attila Forgacs, Ildiko Garai

**Affiliations:** 1 ScanoMed Nuclear Medicine Centers, Nagyerdei krt. 98, Debrecen, H-4032 Hungary; 2 Mediso Ltd., Budapest, Hungary; 3 Medical Imaging Clinic-Nuclear Medicine, Clinical Center, University of Debrecen, Debrecen, Hungary; 4 Ahmanson Translational Imaging Division, Department of Molecular and Medical Pharmacology, David Geffen School of Medicine, University of California at Los Angeles, Los Angeles, USA

**Keywords:** ^18^F-FDG PET/CT, ^99m^Tc-MDP Bone Scan, DICOM Metadata, Protocol compliance, Quality management

## Abstract

**Background:**

Regular and precise inspection of the realization of the local nuclear medicine standard operation procedures (SOPs) is very complex and time-consuming, especially when large amount of patient data is obtained from a wide scale of different scan procedures on a daily basis. DICOM metadata comprise a complete set of data related to the patient and the imaging procedure, and consequently all information necessary to evaluate the compliance with the actual SOP.

**Methods:**

Q-Bot, an automatic DICOM metadata monitoring tool which is capable to verify SOP conformities, was tested for 11 months at two nuclear medicine departments. Relevant parameters, such as patient ID, patient mass and height, injected activity, and uptake time, were investigated in the case of adult ^18^F-FDG whole-body PET/CT and ^99m^Tc-MDP gamma camera bone scans on a daily basis. Q-Bot automatically inspected the actual SOP compliance of these relevant DICOM parameters. Q-Bot graphical user interface (GUI) provided a summary of the outliers in a table format to be investigated by a dedicated technologist. In addition, information related to the error handling was also collected for retrospective analysis of long-term tendencies.

**Results:**

In total, 6702 PET/CT and 2502 gamma camera scans were inspected, from which 8581 were confirmed as valid patient study without errors. Discrepancies related to the lack of a parameter, not appropriate format, or improper scan procedures were found in 623 cases, and 156 out of these were corrected before the medical reading and reporting. SOP non-conformities explored with Q-Bot were found to be non-correctable in 467 cases. Systematic errors to our practice turned out to be the manual radiopharmaceutical injection, the allowance to use both SI and non-SI units, and the clear definition of decimal point symbol to use.

**Conclusion:**

The daily evaluation of Q-Bot results provided early detection of errors and consequently ensured the minimization of error propagation. Integration of a QM software that inspects protocol compliance at a nuclear medicine department provides significant support to detect non-conformities for technologists, and much higher confidence in image quality for physicians.

## Introduction

Quality management (QM) plays an essential role in the nuclear medicine clinical workflow, driven by the desire for high diagnostic accuracy standards. A well-established QM program would minimize the risk of unnecessary patient dosage, secure the optimal image quality, and provide reliable reconstructed image data for quantitative measures. QM has two fundamental components to comply with these purposes, the process of quality assurance (QA) and quality control (QC) tests. While QA should always be a proactive system, aimed to prevent procedural errors and failures of equipment, it needs to regularly observe the systematic processes as well. On the contrary, QC tests have the essential role of identifying unacceptable errors that may occur during normal operations. International standards for medical equipment were introduced for QM purposes [1], as well as recommendations and guidelines became available for the standardization of patient examination protocols for both PET/CT [2, 3] and conventional nuclear medicine techniques [4]. The scope of these guidelines is to comprehensively describe the optimal workflow from the patient registration to the medical report. In accordance with these guidelines, each nuclear medicine center may implement adequate protocols tailored to their own operation. These protocols as part of the local QA include standard operation procedures (SOPs) for the optimal workflow and for the case of unexpected events as well. Harmonization efforts focusing on multicenter trials have increased in the recent years, recommending strict QC phantom measurements to minimize bias in quantitative measurements [5–12]. The occurrence of procedural mistakes can be minimized in the daily routine with standardized protocols; however, by nature, these errors are inevitable. Longitudinal studies aiming to explore temporal consistency highlighted variations in SPECT system stability [13], as well as standardized uptake value (SUV) of ^18^F-FDG PET/CT [14–17], showing fluctuations related to several sources including dose calibrator accuracy [18]. These investigations, however, deal only with instrumentation and are not considering errors related to human sources, inappropriate patient logistics, or instable instrumentation.

Digital Imaging and Communications in Medicine (DICOM) became the most widely used medical image format even in nuclear medicine. DICOM images consist of two parts: (1) the *medical image information* (slices or series of images) and (2) the so-called *metadata or header*, including information about the patient, radiopharmaceutical, scanner settings, and the reconstructed image-related parameters. Several applications have recently been developed that allows for monitoring and retrieving DICOM header information, which makes it possible to generate statistics related to a specific modality or generic features in a modality-independent way [19–22]. Moreover, radiation exposure monitoring of patients using DICOM information was also introduced [23–26] including the possibility to detect irregular radiation doses in the case of CT and digital coronary angiography (DCA) procedures. In addition, highlighted DICOM metadata elements are recommended to be monitored regularly to verify the appropriate use of SUV as an imaging biomarker when considering a successful multicenter PET/CT study [27]. Similar criteria may apply for other widely used quantitative measures such as metabolic tumor volume (MTV) or total lesion glycolysis (TLG). The rationale of longitudinal check of patient scan parameters using the metadata information of DICOM files arises from the need to verify protocol compliance. Hristova et al. [27] reported a list of important DICOM metadata elements containing the parameters, which strongly affect the correct use of imaging biomarkers. The monitoring of these parameters with a user-friendly application is therefore desirable. In this work, we present a software tool named the Quality Management Bot (Q-Bot), which is capable to automatically monitor DICOM metadata and perform evaluations in the aspect of QM focusing on nuclear medicine studies. By using header information retrieved from real patient DICOM files, the presence of errors in the clinical workflow as reflected in the metadata can be detected. On the other hand, all patient examinations which did not include the errors mentioned above are certified. The Q-Bot was introduced in the routine clinical workflow of two nuclear medicine departments and monitored DICOM records for several months. In this paper, we present the most relevant clinical use of Q-Bot that may provide valuable information to other nuclear medicine centers as well.

## Materials and methods

**Fig. 1:**
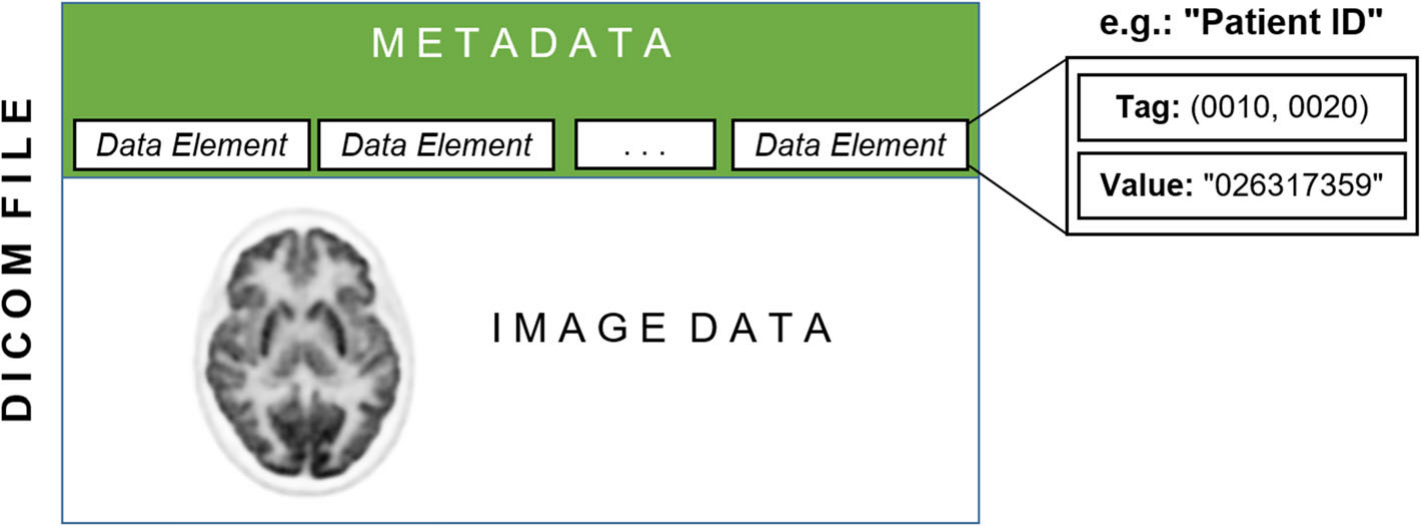
A DICOM file consists of image data and metadata. The metadata is a set of data elements each including identifying DICOM tag and a corresponding value

The metadata in the header of the DICOM images include information about the patient, the applied radiopharmaceutical and its administration, the scanner acquisition settings, and the reconstructed image-related parameters. The metadata regularly includes data elements, and each of them has an identifying code (i.e., DICOM tag) and an actual value as indicated in Fig. 1. The DICOM tag can uniquely identify the corresponding data element. Picture Archiving and Communication System (PACS) servers are available for a user-friendly management and storage of DICOM files. General purpose DICOM clients and browsers provide direct access only to a limited subset of the DICOM metadata.

**Fig. 2:**
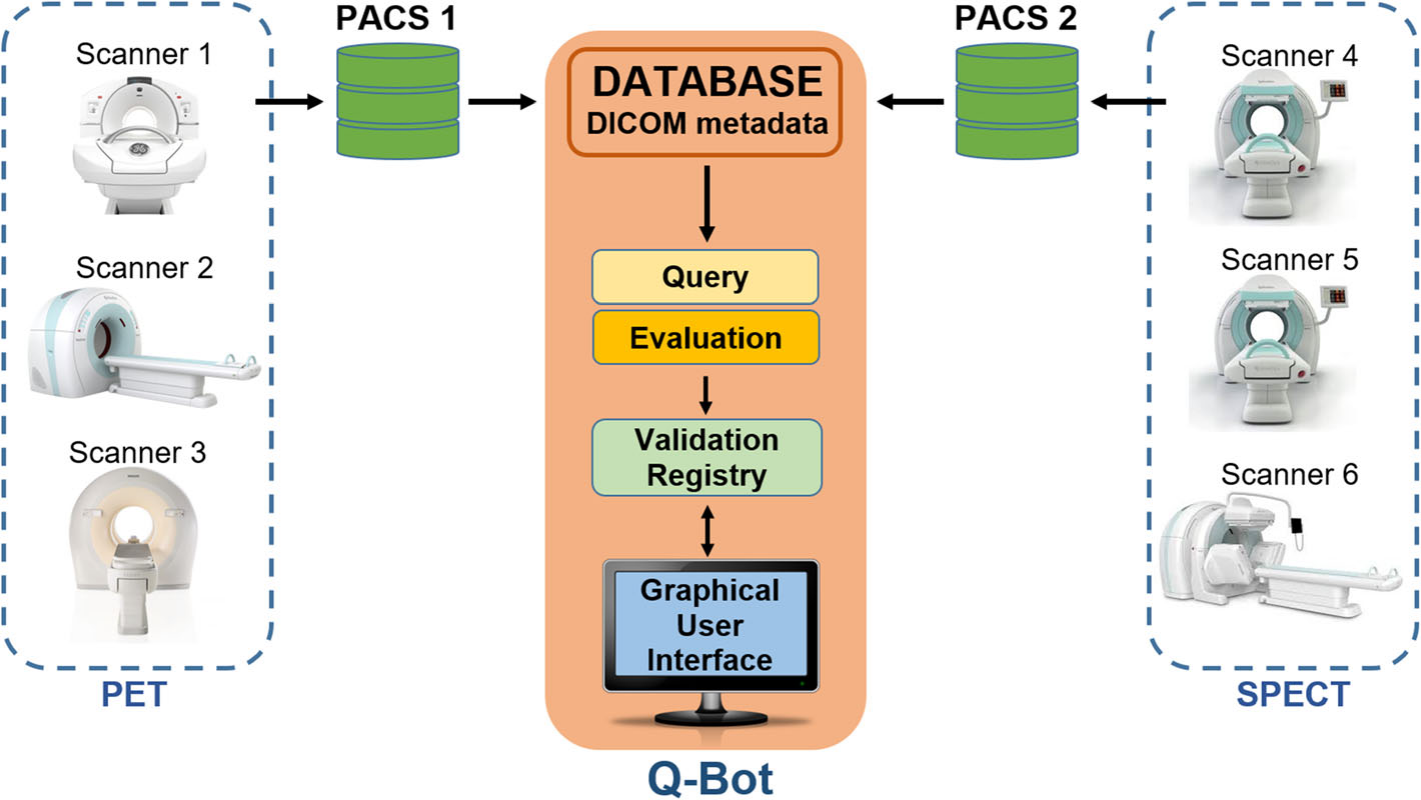
Data flow from the image data acquired on the scanners to the Q-Bot software tool. The Q-Bot consists of five main components: the DICOM tag database, the query module, the evaluation module, the validation registry, and the graphical user interface. The results of the evaluation are recorded in the validation registry, and these records are managed via the user interface

Thus, performing a query on DICOM files stored on a PACS server with a complex filtering condition based on arbitrary DICOM tags is generally not possible. Furthermore, to read the value of a not directly accessible tag, the corresponding DICOM files have to be downloaded before reading. In order to make all DICOM tags directly available for QM monitoring, we used a Non-Structured Query Language (NoSQL) database as a way to handle DICOM metadata originating from various imaging scanners of two clinical sites. This method allowed us to have a direct access to all standard and private tags of DICOM images stored on our PACS servers paving the way to create a QM monitoring tool and perform long-term statistical analysis. In this study, a DICOM tag database was used with the potential of storing all the metadata with arbitrary structure and without any modification. The database was continuously synchronized with PACS data on a regular basis for consistency. Q-Bot monitors DICOM tags in the synchronized database with respect to *patient information* (e.g., patient ID, weight and height, age), *applied radiopharmaceutical* (e.g., radionuclide, injected amount of radioactivity, measurement, and injection time), and *acquisition parameters* (e.g., scan start, time per bed position, acquired counts). The data flow from PET and SPECT scanners to the Q-Bot user interface is shown in Fig. 2. The Q-Bot software consists of five main components: DICOM tag database, query module, evaluation module, validation registry, and a graphical user interface.

### Query module

After creating the DICOM tag database, the query module collects only a set of parameters with regard to the study of interest (also referred to as the data aggregation process) and performs name standardization, since different vendors may call the same study type differently. A configuration file defines this process, and the final result is a simplified and consistent data table, where each row contains primary parameters of a specific study of a single patient. As a next step, the query module extends the output table with further parameters calculated from the primary ones, such as radiopharmaceutical uptake time, injected amount of activity normalized to body weight, or the body mass index (BMI).

### Evaluation module

The evaluation module inspects the output table of the query module row by row using an RStudio script as it follows. Parameters in each row were categorized into three groups: patient-specific, study-specific, and patient- and study-specific parameters. *Patient-specific* quantities included patient ID, height, weight, BMI, and age. In Hungary, the patient ID includes nine digits, where the last number is generated with a specific combination of the first eight numbers (i.e., applying a checksum). Therefore, taking into consideration this ID generation method, the correctness of patient ID was easily monitored. The validity of some patient-specific parameters (e.g., patient height, patient weight) was not directly verifiable; however, we set reasonable limits for derived quantities (e.g., 15 < BMI < 50), and the evaluation gave warning message in case these limits were exceeded. The radiopharmaceutical uptake time fell into the *study-specific* category, while the injected amount of radioactivity and the acquisition time duration were patient- and study-specific parameters. In the case of these two categories, the R script identified the study procedure and the scanner model for each row of the query module output table and called the values of the appropriate parameters stored in the output table. Then, these parameter values were compared to the reference values defined in the actual SOP. Uptake time, injected amount of radioactivity, and the acquisition time duration may have specific values for the actual study type (i.e., bone scintigraphy or ^18^F-FDG whole-body PET/CT). Injected radioactivity and the acquisition time duration vary from scanner to scanner even in case of the same study type. The SOP specifies the injected amount of activity, the name of the acquisition protocol, the expected uptake time, and some further acquisition-specific parameters such as acquisition time per bed position. As an example, the required injected activity for ^18^F-FDG whole-body examination of an adult was defined to be 3.5 MBq/kg for the AnyScan PET/CT at our clinic. In the evaluation step, a subpopulation of the examinations was queried for including only the ^18^F-FDG whole-body examinations of adult patients scanned on the scanner mentioned above. For these patients, the calculated injected activity per body weight had to meet the SOP criteria (3.5 MBq/kg) with a given ± 10% tolerance limit.

### Validation registry

**Fig. 3:**
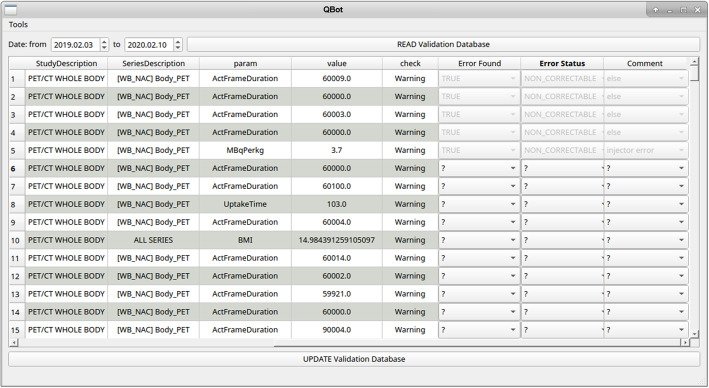
Graphical user interface for listing and supervising records in the validation registry of Q-Bot. The three fields on the right are to be filled out after auditing the actual warning message

As a result of the evaluation, the R script inserted a record for each of the inspected parameters into the validation registry and that consequently appeared on the GUI as can be seen in Fig. 3. One record of the validation registry consists of the parameter name and value, additionally the corresponding study and patient information for the unambiguous error identification. Whether the parameter was in or out of the specified range, a “certified” or “warning” label was added to the record.

### Graphical user interface

The graphical user interface was designed for listing and managing the records of the validation registry as displayed in Fig. 3. This platform helps to get a quick overview of the errors and support the quick identification and response of the problematic examinations. Patient name and patient ID columns have importance to identify the scan; however, these are omitted from Fig. 3 to be compliant with the European Union General Data Protection Regulation [28]. Three extra editable fields are included in each record: “Error Found,” “Error Status,” and “Comment.” These categorical fields are filled by a specially trained or dedicated technologist after the review of the record and the corresponding DICOM metadata. The “error” field is “TRUE” if the detected error was real, otherwise “FALSE” meaning an unjustified warning message. In the “corrected” field, one may select the values “TRUE,” “FALSE,” or “NON_CORRECTABLE.” The “comment” field could be set to predefined categories describing the source of error, such as “human error,” “test measurement,” “camera error,” “injector error,” “minimum dose,” “maximum dose,” “non-reimbursed patient,” “qbot error,” and “else.”

### Clinical use of Q-Bot

The Q-Bot was integrated into our clinical routine in the beginning of the year 2019 at two nuclear medicine departments (ScanoMed Debrecen and ScanoMed Budapest) with three ^18^F-FDG EARL accredited PET/CT systems. One of the two nuclear medicine departments, ScanoMed Debrecen, was European Union of Medical Specialists–European Board of Nuclear Medicine (UEMS-EBNM)-accredited as well. Q-Bot was installed on a dedicated research workstation operated by a technologist with a complete insight into the entire clinical workflow. The patient study validation and error handling process was performed on a daily basis, by inspecting all problematic studies from the examinations of the day before displayed on the GUI. After the inspection of the Q-Bot validation registry, the additional fields (error found, error status, comment) were filled by the dedicated technologist. Since the investigated records were related to the examinations performed a day before, the identifications and corrections were performed prior to the medical reading and reporting by our nuclear medicine specialists. This is due to our usual clinical practice of performing the image interpretation and reading more than 24 h after the scan was performed.

Beyond the daily error management described above, error data compilation was performed on a regular basis. This explorative data analysis of the questionable patient studies was carried out with the RStudio statistical program operating directly on the elements of the validation registry. This task was performed by a medical physicist with experience in data analysis. Task-based query and plots were configured in RStudio, and due to the data analysis and visualization, a more robust and intuitive overview was carried out. The number of deviations from the SOPs was counted, and frequencies were calculated for each parameter as the number of the errors divided by the total number of studies for ^18^F-FDG whole-body PET/CT and ^99m^Tc-MDP gamma camera bone scans respectively. The explorative approach of Q-Bot regularly includes discussions with an experienced nuclear medicine physician or technologist to evaluate and interpret the systematic results.

## Results

### Experiences from the daily evaluation

Nine thousand two hundred four patient scans (6702 PET/CT and 2502 gamma camera) were inspected between the 3 January and 20 November 2019. In total, 8581 scans were confirmed as valid patient study (i.e., no errors found) regarding the patient ID, the BMI (patient weight and height), uptake time, injected activity, and time per bed position (for PET only). The summary of error occurrences in the investigated time period is displayed in Table 1 in accordance with the monitorized parameters for both PET/CT and gamma camera examinations.

**Table 1:** Descriptive statistics of the errors in ^18^F-FDG whole-body PET/CT and ^99m^Tc-MDP bone examinations

Parameter	^**18**^F-FDG whole-body PET/CT		^**99m**^Tc-MDP bone	
	Number	Frequency	Number	Frequency
Total number of studies	6702		2502	
*Injected activity*	102	1.5%	323	11.9%
*Actual frame duration*	39	0.6%	n/a	n/a
*Uptake time*	36	0.5%	51	1.9%
*Patient ID*	31	0.5%	7	0.2%
*BMI*	25	0.4%	9	0.3%
***Total***	**233**	**3.5%**	**390**	**14.3%**

**Fig. 4:**
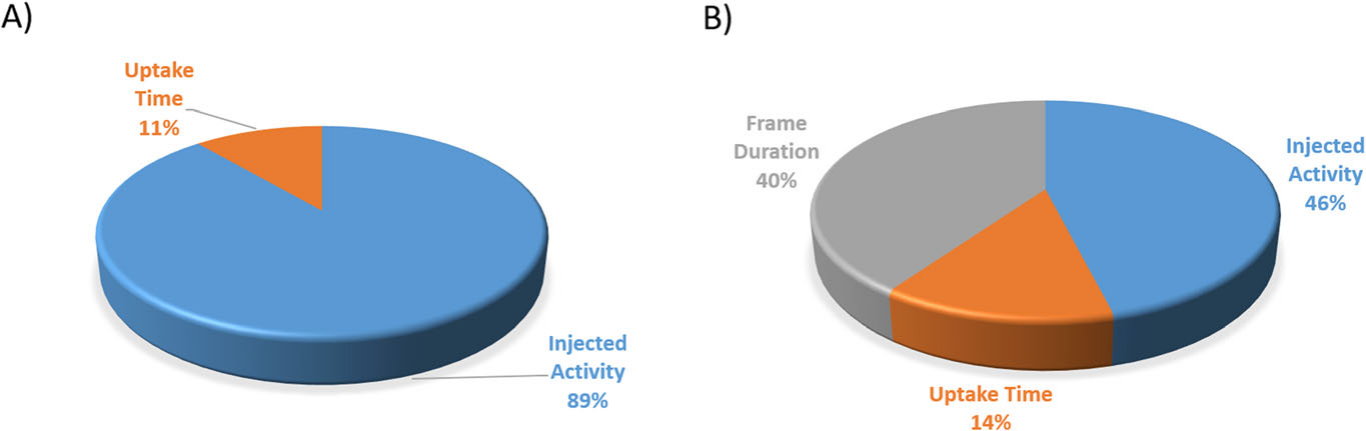
Summary relative frequencies of non-correctable errors in bone scans (**a**)—313 cases—and in ^18^F-FDG whole-body PET-CT (**b**)—154 cases

Errors and discrepancies due to *the lack of a parameter* or *not appropriate format* or *improper scan procedures* were found in 623 cases (233 for PET/CT and 390 for gamma camera). Out of these, 156 errors were corrected before the interpretation and reporting by the nuclear medicine physician. It is important to emphasize that only the errors caused by mistyping can be corrected after the scan. Risk points resulting from these errors in our routine workflow were found to be the parallel appearing SI and non-SI units (kg and lb, mCi and MBq, m, and ft) displayed on the scanner graphical user interface or printed on the patient examination form. In addition, the decimal place symbol (column or point) also infers the potential to introduce errors in the examination routine. Q-Bot explored non-correctable, thus permanent deviations in 467 cases. The relative frequencies of the non-correctable errors are displayed in Fig. 4 for our gamma camera department and two PET departments separately.

### Explorative data analysis

**Fig. 5:**
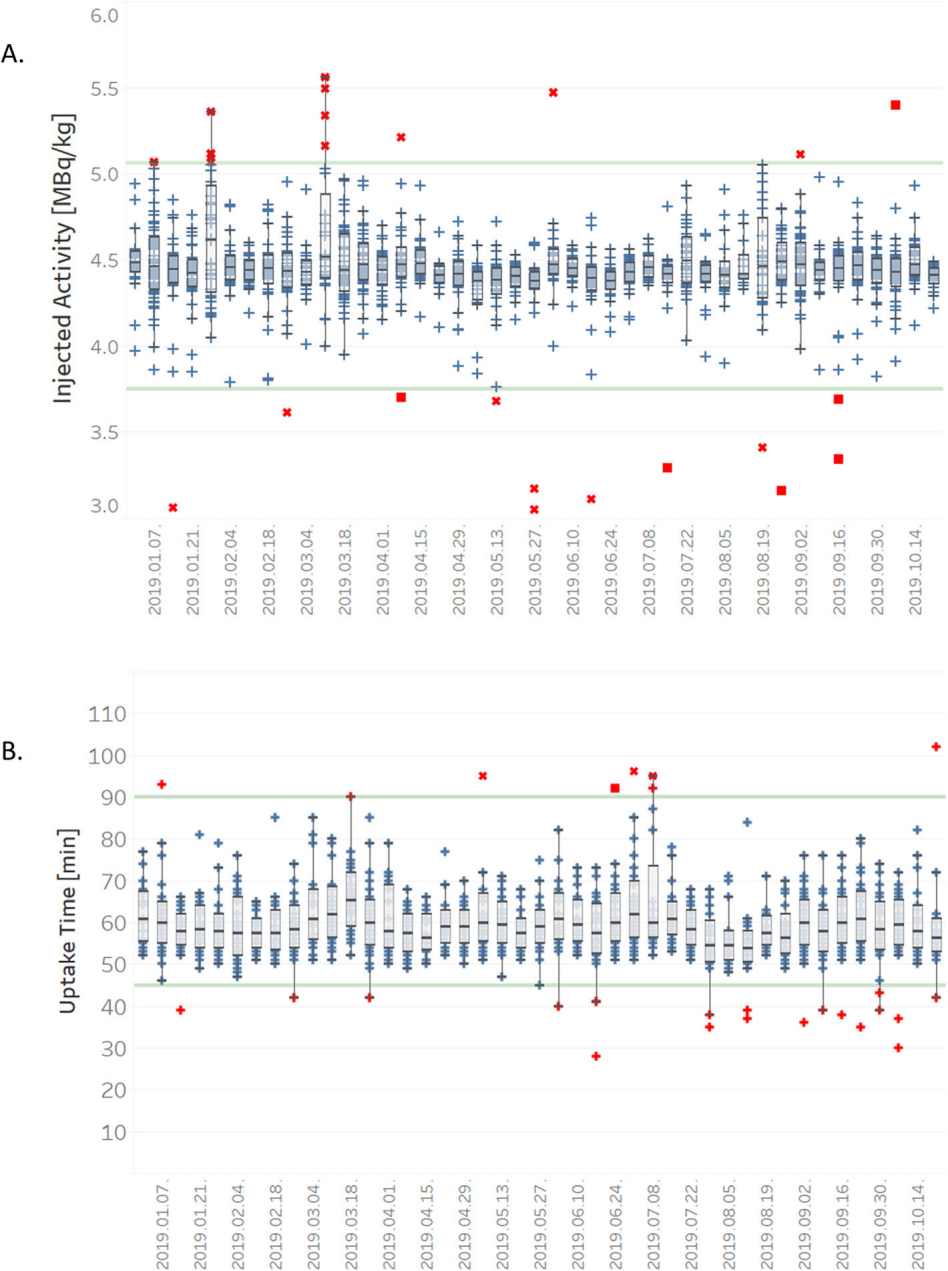
Whisker box plot visualization of injected activity (**a**) and uptake time (**b**) data compiled by the Q-Bot tool for ^18^F-FDG whole-body adult patient scans carried out on one of our PET/CT systems (Philips Gemini 64 TF)

**Fig. 6:**
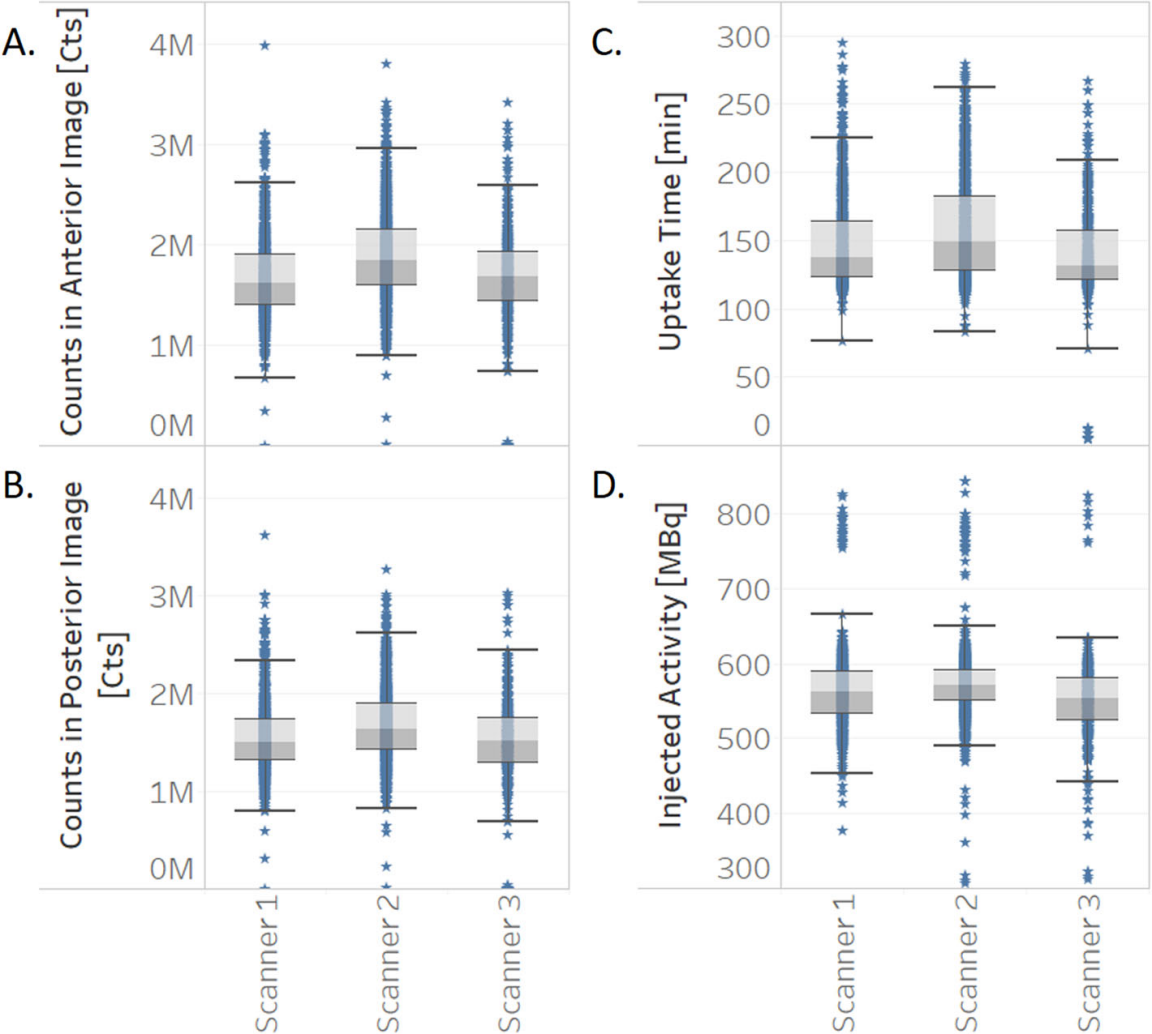
Comparison of three AnyScan SPECT systems from the aspects of total counts of anterior and posterior images acquired (**a**, **b**), injected activity (**d**), and uptake time (**c**) related to ^99m^Tc-MDP bone scans

Beyond the daily DICOM metadata evaluation carried out with the Q-Bot by the dedicated technologist, comprehensive inspection of the database parameters was also performed from the aspects of long-term stability and scanner equivalence. Targeted questions were investigated and even subtle changes were observed. Figure 5a displays the weekly distribution of the amount of injected ^18^F-FDG activity per kilogram body weight in case of whole-body adult patient scans on one of our PET/CT systems (Philips Gemini 64 TF)*.* Whisker box plot visualization of the uptake time can be seen in Fig. 5b together with the SOP-defined limits (green lines) and revealed non-conformities (indicated in red). Comparison of the three SPECT systems from the aspect of counts acquired in case of ^99m^Tc bone scans is displayed in Fig. 6a, b. This figure confirmed the similar sensitivity of our three SPECT scanners. Moreover, in Fig. 6c, d, one may observe that the currently applied patient logistics assure the proper injected activity and uptake time for all three scanners.

## Discussion

QM in nuclear medicine aims to ensure the efficiency and reliability of the clinical practice, including image quality and optimal patient dose. Available international practical guidelines [2, 4] support the formation of local SOPs, adding up to the definition of individual patient examinations. Participation in multicenter studies necessitates additional SOPs resulting in harmonized image quality between imaging sites [5, 7, 8]. Furthermore, the level of QM of nuclear medicine centers can be evaluated (and eventually certified) through advanced clinical accreditation programs provided by international organizations [5–8, 10–12, 29–31]. Even with SOPs tailored well to the relevant regulations and recommendations mentioned above, appropriate QM has to ensure the compliance with these documents as well. The regular and precise inspection of the occurrence of errors and interruptions the nuclear medicine technologists facing in daily clinical practice is very complex and time-consuming [32], especially when large amount of patient data is obtained from a wide scale of different scan procedures on a daily basis. A software tool offering an advanced solution to perform this task would be very beneficial and effective. DICOM headers comprise a near-complete set of data related to the patient and the imaging procedure, and consequently all information necessary to evaluate the compliance with the actual SOP.

In this study, we presented an automatic DICOM metadata monitoring tool, the Q-Bot, which is capable to verify SOP conformities. Q-Bot was initiated at two nuclear medicine clinics in the beginning of January 2019, and since then the software monitorized more than 9000 patient scans including adult ^18^F-FDG whole-body PET/CT and ^99m^Tc-MDP gamma camera bone scans. Relevant parameters, such as patient ID, patient mass and height, injected activity, and uptake time were investigated on the conducted patient scans on a daily basis. The Q-Bot automatically evaluated the DICOM metadata of each patient scan by comparing the appropriate values of the inspected DICOM tags to the reference values defined in the local SOPs. The Q-Bot graphical user interface (GUI) provided a summary of the outliers in a table format (Fig. 3) to be investigated by the dedicated technologist. Moreover, this GUI comprised some extra input fields to collect additional information related to the error handling (error status, source of error, etc.). Both the registered outliers and feedback of technologist were saved in the validation registry, opened a way to retrospectively explore long-term tendencies. The daily evaluation of the Q-Bot results provided early detection of errors and consequently ensured the minimized risk of error propagation. Each problematic patient study was reviewed and corrected or clearly marked prior to the medical reading and reporting, and also prior to sharing the diagnostic images with the patient or with the referring physician.

Mistyped patient ID makes the start of medical reporting complicated due to the extra time necessary to identify the study. Furthermore, if a follow-up study was performed, one of the patient examination series could be easily overlooked by the physician. Typographical entry errors related to the body weight, height, injected activity, and time cause distortion in the quantitative measures (e.g., SUV, MTV, TLG in case of ^18^F-FDG PET studies [14]) and give an impression that the scan was performed inaccurately. These errors can be identified by performing the daily Q-Bot routine study validation. The short- or long-term visualization of the observed deviations from the SOPs helped to discover systematic errors related to critical elements of the clinical workflow. In our practice, manual radiopharmaceutical injection, allowance to use both SI and non-SI units, and omission prescribing the decimal point symbol to use turned out to be characteristic types of systematic errors. The occurrence of these errors can be minimized by appropriate staff training and making patient documentation straightforward. The relative frequencies of errors for each inspected parameter are summarized in Table 1, while Fig. 4 focused only on non-correctable errors in PET and gamma camera studies. The number of errors detected in case of ^99m^Tc-MDP bone scans (Fig. 4a) was much higher than that of ^18^F-FDG whole-body PET scans (Fig. 4b). The reason behind this was related to an error in our SOP that ordered a total injected activity range of 500–600 MBq independently from body weight, and it was erroneously assumed to result in the 5–10 MBq/kg in all cases evaluated/monitorized by Q-Bot.

A representative distribution of injected ^18^F-FDG activity per kilogram body weight over a period of 9 months is displayed in Fig. 5a. It reveals time periods in 2019 (e.g., 2 weeks in March), with higher median values and lower precision compared to other time periods. However, these values still remained within the SOP defined ± 10% tolerance limits (indicated with green lines in Fig. 5a).

These fluctuations were induced by substituting the automatic activity dispenser with manual injection caused by various reasons (e.g., no injector kit supply or not optimal radioactivity concentration of the received ^18^F-FDG stock solution). Box and whisker plot visualization of the uptake time displayed in Fig. 5b together with the SOP defined limits (green lines) also revealed non-conformities (indicated in red). Figure 6 clearly proves the equivalence of three SPECT systems in our laboratory with regard to the total accumulated counts (panels a and b) as well as the injected activity and the time regime of the scans.

As an example, the two most frequently performed procedures (adult ^18^F-FDG whole-body PET/CT and ^99m^Tc-MDP bone scans) were examined in this work. Similarly, the same kind of analysis methodology and visualization (Figs. 5 and 6) would allow documentation of the compliance with the specific local SOP of the data entered in routine nuclear medicine examinations at any laboratory. The same kind of control carried out manually would be extremely time-consuming which underlines the efficiency and power of the Q-Bot. The daily evaluation feature of this tool means a retrospective analysis and offers the possibility to react on revealed warning messages. A more effective solution could draw attention on the fly to inappropriate patient data, scanning parameters, acquired data making possible the immediate response. Operation of the Q-Bot requires standardized protocol names (i.e., study and series descriptions) and a comprehensive set of SOPs.

## Conclusions

The integration of a QM software at a nuclear medicine department inspecting protocol compliance provides significant support for technologists to detect non-conformities, and much higher confidence in image quality for the physicians. For this purpose, we developed Q-Bot an automatic DICOM metadata monitoring tool and demonstrated the application and usefulness of this software in the clinical setting through the examples of some representative study types. Continuous operation of this kind of software would enable the detection of both systematic and random errors. Thus, it would improve diagnostic accuracy in the field of nuclear medicine.

## Data Availability

The datasets supporting the conclusions of this article are available from the corresponding author on reasonable request.

## Abbreviations

BMI: Body mass index
CT: Computed tomography
DCA: Digital coronary angiography
DICOM: Digital Imaging and Communications in Medicine
GUI: Graphical user interface
MTV: Metabolic tumor volume
NoSQL: Non-Structured Query Language
PACS: Picture Archiving and Communication System
PET: Positron emission tomography
QM: Quality management
QA: Quality assurance
QC: Quality control
SPECT: Single-photon emission computed tomography
SOP: Standard operation protocol
SUV: Standardized uptake value
TLG: Total lesion glycolysis

## References

[1] ISO (International Organization for Standard) (2016). ISO 13485: 2016 medical cdevices - quality management system - requirements for reguatory purpose.

[2] Boellaard R, Delgado-Bolton R, Oyen WJG, Giammarile F, Tatsch K, Eschner W (2015). FDG PET/CT: EANM procedure guidelines for tumour imaging: version 2.0. Eur J Nuclear Med Mol Imaging.

[3] International Atomic Energy Agency. IAEA Health Human Series No. 1: Quality Assurance for PET and PET/CT Systems. IAEA Hum Health. 2009;1(1) Available from: http://insights.ovid.com/crossref?an=00004032-201212000-00028.

[4] Van den Wyngaert T, Strobel K, Kampen WU, Kuwert T, van der Bruggen W, Mohan HK, et al. The EANM practice guidelines for bone scintigraphy. Eur J Nucl Med Mol Imaging 2016;43(9):1723–1738. Available from: 10.1007/s00259-016-3415-4 10.1007/s00259-016-3415-4 PMC4932135 27262701

[5] Kaalep A, Sera T, Oyen W, Krause BJ, Chiti A (2018). EANM / EARL FDG-PET / CT accreditation - summary results from the first 200 accredited imaging systems. Eur J Nucl Med Mol Imaging.

[6] Kaalep A, Sera T, Rijnsdorp S, Yaqub M, Talsma A, Lodge MA (2018). Feasibility of state of the art PET/CT systems performance harmonisation. Eur J Nucl Med Mol Imaging.

[7] Aide N, Lasnon C, Veit-haibach P, Sera T, Sattler B, Boellaard R (2017). EANM / EARL harmonization strategies in PET quantification: from daily practice to multicentre oncological studies. Eur J Nucl Med Mol Imaging.

[8] Boellard R (2012). Manual for EARL FDG-PET/CT Accreditation.

[9] MacFarlane CR (2006). ACR accreditation of nuclear medicine and PET imaging departments. J Nucl Med Technol.

[10] Katanick SL (2005). Fundamentals of ICANL accreditation. J Nucl Med Technol.

[11] Intersocietal Accreditation Commission (2018). The IAC Standards and Guidelines for Nuclear/PET Accreditation.

[12] Kaalep A, Burggraaff CN, Pieplenbosch S, Verwer EE, Sera T, Zijlstra J (2019). Quantitative implications of the updated EARL 2019 PET–CT performance standards. EJNMMI Phys.

[13] Anizan N, Wang H, Zhou XC, Hobbs RF, Wahl RL, Frey EC (2014). Factors affecting the stability and repeatability of gamma camera calibration for quantitative imaging applications based on a retrospective review of clinical data. EJNMMI Res.

[14] Lockhart CM, MacDonald LR, Alessio AM, McDougald WA, Doot RK, Kinahan PE (2011). Quantifying and reducing the effect of calibration error on variability of PET/CT standardized uptake value measurements. J Nucl Med.

[15] MacDonald LR, Perkins AE, Tung C-H (2016). Longitudinal monitoring of reconstructed activity concentration on a clinical time-of-flight PET/CT scanner. J Med Imaging.

[16] Byrd D, Christopfel R, Arabasz G, Catana C, Karp J, Lodge MA (2019). Erratum: measuring temporal stability of positron emission tomography standardized uptake value bias using long-lived sources in a multicenter network. J Med Imaging.

[17] Doot RK, Pierce LA, Byrd D, Elston B, Allberg KC, Kinahan PE (2014). Biases in multicenter longitudinal PET standardized uptake value measurements. Transl Oncol.

[18] Mcdougald WA, Miyaoka RS, Alessio AM, Harrison RL, Lewellen TK. A study of SPECT/CT camera stability for quantitative imaging. EJNMMI Phys 2016;3(14):1–13. Available from: 10.1186/s40658-016-0150-7 10.1186/s40658-016-0150-7 PMC4967048 27473290

[19] Valente F, Costa C, Silva A (2013). Dicoogle, a Pacs featuring profiled content based image retrieval. PLoS One.

[20] Santos M, Bastião L, Costa C, Silva A, Rocha N (2011). DICOM and clinical data mining in a small hospital PACS: a pilot study. Commun Comput Inf Sci.

[21] Langer SG. A flexible database architecture for mining DICOM objects: the DICOM Data Warehouse. J Digit Imaging 2012;25(2):206–212. [cited 2019 Jan 29], Available from: 10.1007/s10278-011-9434-6 10.1007/s10278-011-9434-6 PMC3295972 22080292

[22] Langer SG. DICOM Data Warehouse: Part 2. J Digit Imaging 2016;29(3):309–313. Available from: 10.1007/s10278-015-9830-4 10.1007/s10278-015-9830-4 PMC4879026 26518194

[23] Bastiaõ Silva LA, Ribeiro LS, Santos M, Costa C, Oliveira JL (2014). Screening radiation exposure for quality assurance. Stud Health Technol Inform.

[24] Boos J, Meineke A, Bethge OT, Antoch G, Kröpil P (2016). Dose monitoring in radiology departments: status quo and future perspectives. RoFo Fortschritte auf dem Gebiet der Rontgenstrahlen und der Bildgeb Verfahren.

[25] Boos J, Meineke A, Rubbert C, Heusch P, Lanzman RS, Aissa J (2016). Cloud-based CT dose monitoring using the DICOM- structured report: fully automated analysis in regard to national diagnostic reference levels. RoFo Fortschritte auf dem Gebiet der Rontgenstrahlen und der Bildgeb Verfahren.

[26] Al-Jabri AJ, Alzimami K, Alsafi K, Alaamer AS, Al-Rajhi MA, Suliman II (2019). Retrospective analysis of patient radiation doses in digital coronary angiography and interventions. Radiat Prot Dosimetry.

[27] Hristova I, Boellaard R, Galette P, Shankar LK, Liu Y, Stroobants S (2017). Guidelines for quality control of PET/CT scans in a multicenter clinical study. EJNMMI Phys.

[28] European Parlament and European Council. REGULATION (EU) 2016/679 of the European Parliament and of the Council of 27 April 2016 on the protection of natural persons with regard to the processing of personal data and on the free movement of such data, and repealing Directive 95/46/EC (General Da. Off J Eur Union. 2016;2014(March 2014).

[29] Dondi M, Torres L, Marengo M, Massardo T, Mishani E, Van Zyl EA (2017). Comprehensive auditing in nuclear medicine through the International Atomic Energy Agency Quality Management Audits in Nuclear Medicine (QUANUM) Program. Part 1: the QUANUM Program and Methodology. Semin Nucl Med.

[30] Dondi M, Torres L, Marengo M, Massardo T, Mishani E, Van Zyl EA (2017). Comprehensive auditing in nuclear medicine through the International Atomic Energy Agency Quality Management Audits in Nuclear Medicine Program. Part 2: Analysis of results. Semin Nucl Med.

[31] Dondi M, Paez D, Torres L, Marengo M, Delaloye AB, Solanki K (2018). Implementation of quality systems in nuclear medicine: why it matters. An Outcome Analysis (Quality Management Audits in Nuclear Medicine Part III). Semin Nucl Med.

[32] Larcos G, Prgomet M, Georgiou A, Westbrook J (2017). A work observation study of nuclear medicine technologists: interruptions, resilience and implications for patient safety. BMJ Qual Saf.

